# Molecular Characterization of Coxsackievirus B5 Isolates from Sewage, Italy 2016–2017

**DOI:** 10.1007/s12560-019-09395-z

**Published:** 2019-07-26

**Authors:** Stefano Fontana, Stefano Fiore, Gabriele Buttinelli, Concetta Amato, Licia Veronesi, Roberta Zoni, Maria Triassi, Francesca Pennino, Giovanni Maurizio Giammanco, Simona De Grazia, Antonella Cicala, Angelo Siragusa, Sabine Gamper, Silvia Spertini, Paolo Castiglia, Andrea Cossu, Cinzia Germinario, Angela Maria Vittoria Larocca, Paola Stefanelli

**Affiliations:** 1 Department of Infectious Diseases, Italian National Institute of Health (Istituto Superiore di Sanità, ISS), Viale Regina Elena 299, 00161 Rome, Italy; 2 Department of Medicine and Surgery, University of Parma, Parma, Italy; 3 Department of Public Health, University of Naples Federico II, Naples, Italy; 4 Department of Health Promotion, Mother and Child Care and Internal Medicine ‘G. D’Alessandro’, University of Palermo, Palermo, Italy; 5 AMAP S.p.A, Palermo, Italy; 6 Comprensorio Sanitario di Bolzano, Servizio Igiene e Sanità Pubblica, Bolzano, Italy; 7 Department of Medical, Surgical and Experimental Sciences, University of Sassari, Sassari, Italy; 8 Department of Biomedical Science and Human Oncology, University of Bari “Aldo Moro”, Bari, Italy

**Keywords:** Coxsackievirus, CV-B5, Sewage, Non-polio enteroviruses, Phylogenetic analysis, Polioviruses

## Abstract

Hereby, the partial Viral Protein 1 sequences of Coxsackievirus B5 (CV-B5) from sewage samples, collected in Italy from 2016 to 2017, were compared with those available in GenBank from clinical samples. Phylogenetic analysis highlighted: (I) the predominant circulation of CV-B5 genogroup B in Italy, and (II) the presence of two new sub-genogroups.

## Introduction

Environmental surveillance (ES) provides an early warning system for a possible introduction of poliovirus and, since 1996, is one of the activities of the Italian WHO Collaborative Reference and Research Center for Polio (2015). Meanwhile, ES examines the circulation and the spatio-temporal distribution of non-polio enteroviruses (NPEVs; Pons-Salort et al. 2018). In a recent study, our group analyzed more than 2800 sewage samples collected from 2009 to 2015. More than half of the samples were positive for NPEVs and Coxsackievirus B5 (CV-B5) being the most frequent serotype (Delogu et al. 2018).

Coxsackie B viruses are frequently associated with sporadic cases of neurological diseases, epidemics of meningitis, and chronic diseases such as cardiomyopathy and diabetes (Tracy and Gauntt 2008; Wikswo et al. 2009; Liu et al. 2014; Tao et al. 2014; Ma et al. 2013; Yao et al. 2017).

Henquell et al. (2013) described the genetic diversity of human CV-B5 clinical isolates with two main genogroups, A and B, detected worldwide. Genogroup A is characterized by sequential acquisition of nonsynonymous changes in residues exposed at the virus 5-fold axis; genogroup B is marked by the selection of three changes in the VP1 C-terminus from its first emergence.

The main aim of this study was to type the NPEVs identified from sewage samples collected from 2016 to 2017 in Italy and to compare the partial VP1 target gene of Italian CV-B5 strains in order to determine their sub-grouping.

## Materials and Methods

Sewage samples were collected from 17 inlets of wastewater treatment plants (WWTPs) serving the urban areas of Naples, Bolzano, Parma, Sassari, Bari, Palermo, Catania, Messina, Trapani, and Syracuse from 2016 to 2017. All samples, except those from Parma, were sent to the WHO collaborative center at the Istituto Superiore di Sanità (ISS), Rome, and were analyzed for the presence of Polioviruses and NPEVs. Samples from Parma were analyzed locally by the Sub-National Polio Reference Laboratory at the University of Parma. Molecular characterization of all polioviruses and NPEVs was performed at the ISS. Wastewater sampling and virus concentration were performed according the WHO Guidelines (2015). Briefly, wastewater samples were collected at the inlet collector, before treatment, and then concentrated by the two-phase separation method [polyethylene glycol (PEG)–dextran] obtaining an approximately 50-fold volume reduction. Seven WWTPs had a population equivalent greater than 300,000. At least 1 sample every 15 days for WWTPs serving > 300,000 inhabitants and at least 1 sample every month for populations of < 300,000 have been taken. An automatic 24 h sampling system was present in four inlets located in Napoli, Palermo, and Bolzano. Manual sampling was performed early in the morning (peak hours) in the remaining cities. Concentrated sewage was inoculated both on RD (human rhabdomyosarcoma cells) and on L20B (genetically modified murine cell line L-series) cell monolayers and analyzed for poliovirus and NPEVs, according to the WHO algorithm (2015).

Viral RNAs were extracted from 200 µl of cell lysate from samples with cellular cytopathic effect using Viral Nucleic Acid Extraction Kit II (Geneaid, New Taipei, Taiwan), according to the manufacturer’s instruction. RT-nested-PCR was performed as previously described with slight modifications: the first amplification step was performed with 222/224 oligonucleotides and the second with AN88/AN89 (Nix et al. 2006; CDC–WHO 2015). Briefly, the first PCR round was carried out using the Access RT-PCR System kit (Promega, Madison, Wisconsin, USA) with an initial reverse transcription step at 45 °C for 40 min, followed by 94 °C for 2 min, 40 cycles at 94 °C for 30 s, 42 °C for 45 s, 68 °C for 60 s, and a final extension at 72 °C for 5 min. Second round was performed using GOTAQ Green 2X Master G2 kit (Fisher Molecular Biology, Rome, Italy) at the following conditions: 95 °C for 30 s, followed by 40 cycles at 95 °C for 30 s, 60 °C for 20 s, 72 °C 30 s, and a final extension for 5 min at 72 °C. GeneAmp^®^ PCR System 9700 thermal cycler (Applied Biosystems, Inc., Foster City, CA) was used for both rounds. The final amplification products, separated on 1.2% agarose gel stained with GelRed^®^ (Biotium, Fremont, California, USA), were inspected with Molecular Imager^®^ Gel Doc™ XR using the Quantity-One^®^ software (BioRad, Segrate, Italy). Sanger nucleotide sequencing of partial VP1 gene (319 nucleotides) was also performed using the AN88/AN89 primers on the nested-PCR products. Sequence analysis and comparison was achieved with software Sequencer^®^ 5.2 (Gene Codes Corporation, Ann Arbor, Michigan, USA) and NCBI GenBank (http://www.ncbi.nlm.nih.gov). The sequences were aligned together with 11 VP1 sequences of CV-B5 genogroups A and 9 of CV-B5 of genogroup B published by Henquell et al. (2013). A phylogenetic tree, based on the 319 nucleotides of the VP1 region, was constructed using MEGA6 software (www.megasoftware.net) following the maximum likelihood method (Kimura 2-parameter model, gamma distributed).

## Results

Overall, 423 sewage samples were collected, of which 244 were NPEV-positive by the cellular cytopathic effect on the RD cell line.

Half of the NPEV-positive samples (122/244) were selected for viral typing. In particular, for each Italian city participating in the surveillance we selected, in the period (2016–2017), half of their NPEVs positive samples. The most frequent genotype was CV-B5 (26.2%, 32/122), followed by Echovirus (E)-6 (22.10%, 27/122), E-11 (12.30%; 15/122), and CV-B3 (11.5%, 14/122). The remaining 34 isolates belonged to 10 different genotypes: E-13 (7.38%), CVB-B4 (5.74%), E-25 (4.92%), E-7 (4.10%), E-3 (1.64%), E-30 (0.82%), CVB-B2 (0.82%), E-9 (0.82%), E-20 (0.82%), and E-19 (0.82%). One Sabin-like poliovirus type 3 strain was isolated from the WWTPs plant serving the urban area of Parma in October 2017.

Partial VP1 sequences (nt 2556 to 2874 of CV-B5 strain Faulkner complete genome) from 32 Italian CV-B5 strains, identified in sewage concentrates, were compared with 20 VP1 sequences representative of the 8 CV-B5 sub-genogroups described by Henquell et al. from clinical samples, available in GenBank (https://www.ncbi.nlm.nih.gov/genbank/), from 10 countries over a long time period (1977–2009, Table 1).

**Table 1:** Details of the CV-B5 Viral Protein 1 sequences used in the study

ID	Accession number	Genogroup/sub-genogroup	Type of sample	Country of origin	City of isolation	Year of isolation	Month of isolation	Number of sampling per months
*CF807S*	*HF948028*	*A0*	*Clinical*	*FRA*	*Not reported*	*1977*	*Not reported*	*Not applicable*
*CF595*	*HF948121*	*A1*	*Clinical*	*FRA*	*Not reported*	*1999*	*Not reported*	*Not applicable*
*17036*	*GU300063*	*A1*	*Clinical*	*NLD*	*Not reported*	*1996*	*Not reported*	*Not applicable*
*STU108*	*HF948077*	*A1*	*Clinical*	*DEU*	*Not reported*	*2004*	*Not reported*	*Not applicable*
*P028*	*GU300060*	*A2*	*Clinical*	*PAK*	*Not reported*	*1990*	*Not reported*	*Not applicable*
*CF219051*	*HF948037*	*A3*	*Clinical*	*FRA*	*Not reported*	*2006*	*Not reported*	*Not applicable*
*LIM004*	*HF948229*	*A3*	*Clinical*	*CYP*	*Not reported*	*1996*	*Not reported*	*Not applicable*
*GRE447*	*HF948173*	*A3*	*Clinical*	*FRA*	*Not reported*	*2003*	*Not reported*	*Not applicable*
*CF186106*	*HF948132*	*A4*	*Clinical*	*FRA*	*Not reported*	*2005*	*Not reported*	*Not applicable*
*COPT11098*	*HF948070*	*A4*	*Clinical*	*DNK*	*Not reported*	*2008*	*Not reported*	*Not applicable*
*YZ032*	*GQ246515*	*A4*	*Clinical*	*CHN*	*Not reported*	*2005*	*Not reported*	*Not applicable*
*CF641*	*HF948115*	*B0*	*Clinical*	*FRA*	*Not reported*	*1979*	*Not reported*	*Not applicable*
*614*	*GU300052*	*B0*	*Clinical*	*FIN*	*Not reported*	*1984*	*Not reported*	*Not applicable*
*3939*	*GU300050*	*B0*	*Clinical*	*USA*	*Not reported*	*1982*	*Not reported*	*Not applicable*
*BES1550*	*HF948149*	*B1*	*Clinical*	*FRA*	*Not reported*	*2000*	*Not reported*	*Not applicable*
*119229*	*FJ868290*	*B1*	*Clinical*	*AUS*	*Not reported*	*1992*	*Not reported*	*Not applicable*
*COPT50075*	*HF948263*	*B1*	*Clinical*	*DNK*	*Not reported*	*1993*	*Not reported*	*Not applicable*
*BOL36*	*HF948065*	*B2*	*Clinical*	*ITA*	*Not reported*	*2008*	*Not reported*	*Not applicable*
*NIC001*	*HF948245*	*B2*	*Clinical*	*CYP*	*Not reported*	*2009*	*Not reported*	*Not applicable*
*STU6*	*HF948275*	*B2*	*Clinical*	*DEU*	*Not reported*	*2009*	*Not reported*	*Not applicable*
BZ-16-32	MK517444	B4	Environmental	ITA	Bolzano	2016	September	2
BZ-16-36	MK517473	B4	Environmental	ITA	Bolzano	2016	November	2
BZ-16-45	MK517445	B3	Environmental	ITA	Bolzano	2016	December	2
BZ-17-02	MK517446	B3	Environmental	ITA	Bolzano	2017	January	2
BZ-17-11	MK517443	B4	Environmental	ITA	Bolzano	2017	March	2
BZ-17-23	MK517447	B4	Environmental	ITA	Bolzano	2017	June	2
1CAI-17-01	MK517470	B3	Environmental	ITA	Catania	2017	June	2
1CAI17-02	MK517448	B3	Environmental	ITA	Catania	2017	June	2
1CAI-17-03	MK517449	B3	Environmental	ITA	Catania	2017	August	2
1CAI-17-04	MK517450	B3	Environmental	ITA	Catania	2017	July	2
1CAI-17-06	MK517451	B3	Environmental	ITA	Catania	2017	July	2
2CAI-17-25	MK517452	B3	Environmental	ITA	Catania	2017	September	2
2CAI-17-27	MK517453	B4	Environmental	ITA	Catania	2017	October	2
E276	MK517454	B4	Environmental	ITA	Parma	2017	December	2
E277	MK517455	B4	Environmental	ITA	Parma	2017	December	2
E278	MK517457	B4	Environmental	ITA	Parma	2017	January	2
E279	MK517458	B3	Environmental	ITA	Parma	2017	January	2
O277	MK517456	B4	Environmental	ITA	Parma	2017	December	2
O278	MK517464	B4	Environmental	ITA	Parma	2017	January	2
E281	MK517459	B3	Environmental	ITA	Parma	2017	February	2
1NA-16-18	MK517471	B4	Environmental	ITA	Napoli	2016	February	3
2NA-16-21	MK517472	A4	Environmental	ITA	Napoli	2016	February	2
1NA-16-23	MK517460	B4	Environmental	ITA	Napoli	2016	February	3
2NA-16-28	MK517461	A4	Environmental	ITA	Napoli	2016	March	2
1NA-16-29	MK517474	B4	Environmental	ITA	Napoli	2016	March	3
1NA-17-50	MK517462	B3	Environmental	ITA	Napoli	2017	June	3
1NA-17-58	MK517463	B3	Environmental	ITA	Napoli	2017	February	3
2PA-16-79	MK517465	B3	Environmental	ITA	Palermo	2016	December	2
1PA-17-06	MK517466	B3	Environmental	ITA	Palermo	2017	January	2
2PA-17-10	MK517467	B4	Environmental	ITA	Palermo	2017	February	2
3PA-17-20	MK517468	B3	Environmental	ITA	Palermo	2017	March	1
SS-17-06	MK517469	B4	Environmental	ITA	Sassari	2017	March	2

In italics the data published by Henquell et al. (2013)

**Fig. 1:**
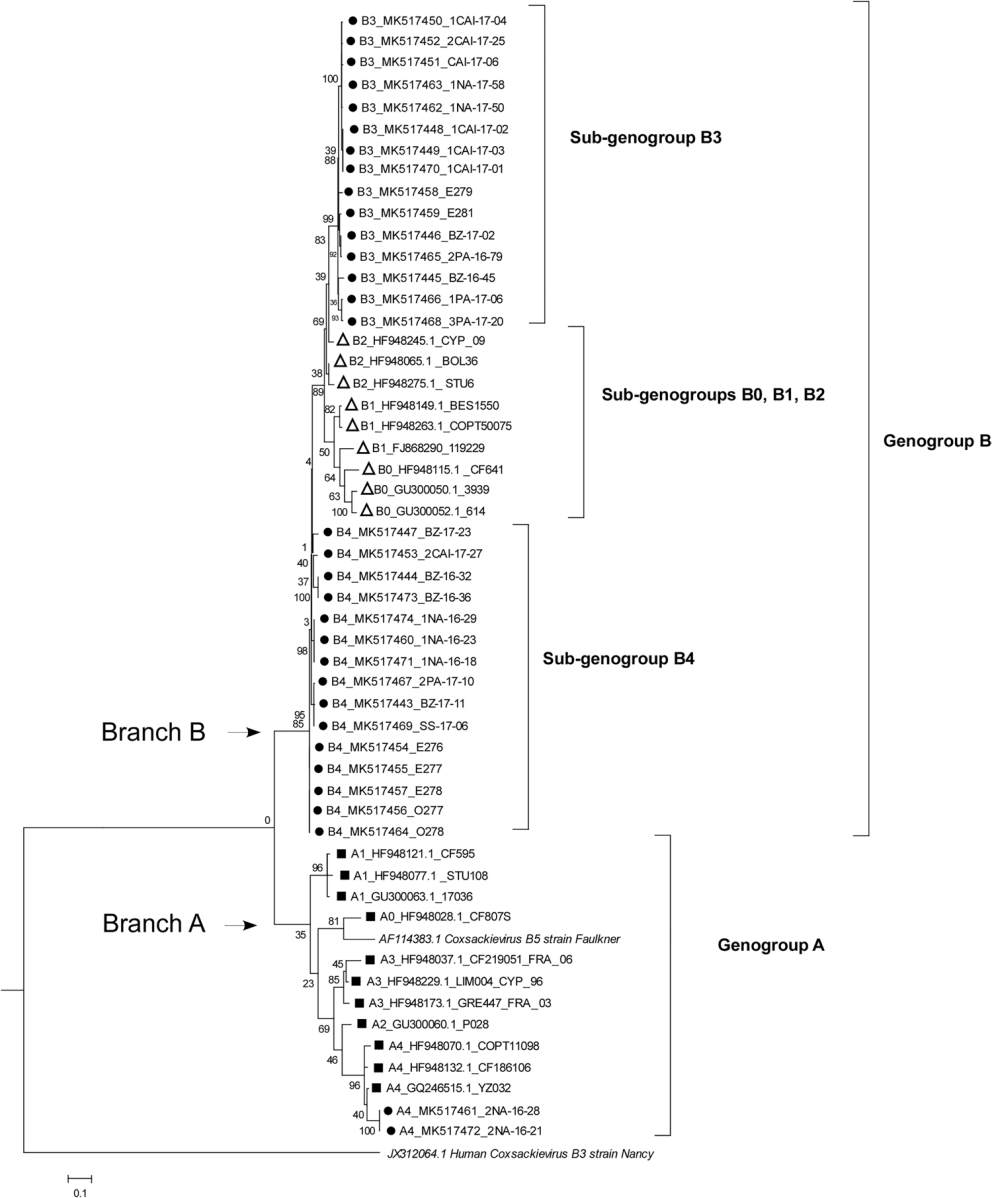
Phylogenetic tree based on the partial VP1 (nt 2556 to 2874 of CV-B5 strain Faulkner complete genome) nucleotide sequences. Trees were built using the maximum likelihood method (Kimura 2-parameter), and bootstrapped with 100 repetitions. Filled circles Italian sewages samples, open triangles genogroup B clinical samples described by Henquell et al. (2013), open squares genogroup A clinical samples described by Henquell et al. (2013)

Figure 1 shows the genetic relationship among 52 VP1 sequences; moreover, the sequences of CV-B5 Faulkner and CV-B3 reference strains were also included.

Two VP1 Italian CV-B5 sequences, from sewage samples in the urban area of Naples, grouped with VP1 CV-B5 Faulkner reference strain within the genogroup A, being similar to the sub-genogroup A4 (Fig. 1). The remaining 30 Italian VP1 sequences, in the B branch together with VP1 sequences of genogroup B CV-B5 strains by Henquell et al., splitted into two novel sub-groups (B3 and B4). In fact, the genetic distance between the two newly described CV-B5 sub-groups (Italian samples) was estimated at 12.3%; while, B3 and B4 sub-groups differed from the sub-genogroups B described by Henquell et al. (sub-genogroups B0, B1 and B2) for 15.2 to 9.6 and for 15.5 to 9.3%, respectively. As a reference, the distance among sub-genogroups B described by Henquell et al. ranged from 6.9 to 13.1%. No relationships were found between the novel B sub-groups and geographic location of the sewage samples.

## Discussion

ES, which is critical to support the global polio eradication endgame, permit to provide early detection of human enteric pathogens excreted with stools during an infection. Several studies reported a clear correlation between the isolation of enteroviruses in sewage, the isolation in humans, and clinical cases identified in the community (Nelson et al. 1967; Manor et al. 1999; Bisseux et al. 2018). All the NPEVs, here described, belonged to the species B, in agreement with what already found in sewage samples collected in Europe (Majumdar et al. 2018). Of note, it is the routine use of RD cell lines that follow the WHO protocol (2015), which favor for the isolation mainly of the EV species B (Majumdar et al. 2018).

The partial sequencing of VP1 was used to determine the serotype and to genetically analyze CV-B5 Italian strains detected in sewages versus CV-B5 strains from clinical samples (Henquell et al. 2013).

The phylogenetic analysis of a 319 nucleotides fragment of VP1 revealed a predominant circulation of genogroup B CV-B5 strains in Italy. This genogroup showed a low rate of evolution in the antigenic determinants over the last 50 years (Henquell et al. 2013).

However, phylogenetic analysis segregated the genogroup B Italian sequences into two relatively distant sub-groups. The marked genetic divergence between the two Italian sequence-clusters and each of the three previously described sub-genogroups, suggests us to consider them as two novel CV-B5 sub-genogroups, namely B3 and B4. Due to the short sampling time period and high genetic conservation of VP1 region, the Italian CV-B5 sequences within sub-genogroups B3 and B4 resulted very similar with a low genetic distance (from 0.00 to 4.00%). In some cases (e.g., IDs E276, E277, E278) the VP1 sequences of the samples collected at the same site and at a short distance of time in the sampling were identical.

Hereby, the main findings are in agreement with what already described in Italy (Delogu et al.) in a more comprehensive sample size collected from 2009 to 2015. Moreover, the predominant circulation of CV-B5 of genogroup B was characterized by the presence of new sub-groups evolving or being recently introduced in Italy.

As in many other European countries, also in Italy the real burden of EV disease can’t be affordably calculated due to many factors including viral diagnosis not always available for central nervous system diseases, pericarditis or cardiomyopathy, and for many other diseases like hand-foot-and-mouth disease or herpangina. Our results emphasize the need for improving national EV surveillance including genetic characterization of the virus isolated in Italy.
